# Supplementary material for: Chromothripsis during telomere crisis is independent of NHEJ, and consistent with a replicative origin
Source: Genome Res. 2019 May;29(5):737–49. doi: 10.1101/gr.240705.118 (PMC6499312; doi:10.1101/gr.240705.118)
Supplement: Supplemental Material [file supp_gr.240705.118_Supplemental_file_1.zip › contigs/annotated_contigs/DB79/contig.2.DB79_length_370_mean_cov_11.5432432432.docx]

**DB79_length_370_mean_cov_11.5432432432**

```
GAAACCTCCGCCTCCTGGGTTCAAACAATTCTCCTGCCTGAGCCTCTCAAGTAGCTGAGATTAGAGACATGCGCCACCACACCCAGCTA
 >chr6:150013887-150014023 + E=2e-67 p=0e+00
ATTTTGTATTTTTAGTAGAGACGAGGTTTCTTCATGTTGGTCTGGCT|CA|TACAGGTGCCCGCCACTATGCCCGGCTAATTTTTTGTA
                                                  >chr6:150014874-150015106 - E=3e-122
TTTTTAGTAGAGACGGGGTTTCACCATGTTGGCCAGGCTGGTCTTGAACTCCTGACCTCAAGTGATCTGCCTGCCTCAGCCTCCCAAAG

TGCTGGGATTACAGGCGTGAGCCACCGCGCCCAGCTAGTAGTACCAATTTATATTCTCCCTATCCGTTTACTATGAGAATGCCTATGTT

TGTAAAGCCTCGGTAT
```
